# Measurement of Heart Rate Variability to Assess Pain in Sedated Critically Ill Patients: A Prospective Observational Study

**DOI:** 10.1371/journal.pone.0147720

**Published:** 2016-01-25

**Authors:** Céline Broucqsault-Dédrie, Julien De Jonckheere, Mathieu Jeanne, Saad Nseir

**Affiliations:** 1 Intensive Care Unit, Hôpital Victor Provo, 35 rue de Barbieux - CS 60359 - 59056 Roubaix Cedex, France; 2 CHU Lille, Clinical Investigation Center - Innovative Technologies, INSERM CIC-IT 1403, F-59000 Lille, France; 3 CHU Lille, Anesthesia and Surgical Critical Care Department, F-59000 Lille, France; 4 CHU Lille, Critical Care Center, F-59000 Lille, France; 5 Univ. Lille, Medicine School, F-59000 Lille, France; Scientific Inst. S. Raffaele Hosp., ITALY

## Abstract

**Introduction:**

The analgesia nociception index (ANI) assesses the relative parasympathetic tone as a surrogate for antinociception/nociception balance in sedated patients. The aim of this study is to determine the effectiveness of ANI in detecting pain in deeply sedated critically ill patients.

**Methods:**

This prospective observational study was performed in two medical ICUs. All patients receiving invasive mechanical ventilation and deep sedation were eligible. In all patients, heart rate and ANI were continuously recorded using the Physiodoloris^®^ device during 5 minutes at rest (T1), during a painful stimulus (T2), and during 5 minutes after the end of the painful stimulus (T3). The chosen painful stimulus was patient turning for washstand. Pain was evaluated at T2, using the behavioral pain scale (BPS). The primary objective was to determine the effectiveness of ANI in detecting pain. Secondary objectives included the impact of norepinephrine on the effectiveness of ANI in detecting pain, and the correlation between ANI and BPS.

**Results:**

Forty-one patients were included. ANI was significantly lower at T2 (Med (IQR) 69(55–78)) compared with T1 (85(67–96), p<0.0001), or T3 (81(63–89), p<0.0001). Similar results were found in the subgroups of patients with (n = 21) or without (n = 20) norepinephrine. ANI values were significantly higher in patients with norepinephrine compared with those without norepinephrine at T1, and T2. No significant correlation was found between ANI and BPS at T2.

**Conclusions:**

ANI is effective in detecting pain in deeply sedated critically ill patients, including those patients treated with norepinephrine. No significant correlation was found between ANI and BPS.

## Introduction

Unfortunately, pain is still a frequent event in critically ill patients. Its incidence is difficult to assess, but at least 50% of surgical or medical patients experience pain during their ICU stay [1]. Chest tube removal, wound drain removal, arterial line insertion, and turning are the most painful procedures performed in the ICU [2]. Pain has a negative impact on patient outcome, and is associated with sleep disturbances, psychological stress and agitation [3]. Further, acute stress response, resulting from pain, includes neurovegetative system and neuroendocrine secretion dysfunctions [4,5].

Recent guidelines on the management of pain, agitation and delirium in adult patients recommend a systemic and rigorous evaluation of pain in critically ill patients, particularly because pain is consistently undertreated in this population [6]. Whilst evaluation of pain could be helpful in improving patient comfort and avoiding over sedation, this could be a difficult task in sedated non-communicative critically ill patients. Behavioral pain scale (BPS), and critical care pain observation tool (CPOT) provide acceptable levels of validity and reliability, and are recommended for nonverbal pain screening. However, these scores have some limitations, including the inter-rater variability, their impossible use in patients receiving neuromuscular-blocking agents, and the discontinuous assessment of pain [7]. In addition, these scores take into account only the physical component of the pain, and do not determine the level of anxiety and discomfort [8].

Analysis of heart rate variability (HRV) is a noninvasive method to evaluate autonomic nervous system (ANS) activity. Heart rate (HR) low-frequency variations, between 0.04 and 0.15 Hz, are related to sympathetic and parasympathetic tones modulations. On the other hand, HR high-frequency variations, between 0.15 Hz and 0.4 Hz, are only related to the parasympathetic tone, which is mainly influenced by respiratory sinus arrhythmia [9,10]. As pain impacts ANS activity, HRV analysis provides a useful surrogate for pain evaluation. Previous studies have shown that pain, fear or anxiety reduce the parasympathetic activity, which can be measured as a decrease of HRV high-frequency spectrum [11–13].

The Analgesia Nociception Index (ANI) device (Physiodoloris^®^, MDoloris Medical Systems, Loos, France) allows noninvasive HRV analysis, by computing the ANI which is related to the parasympathetic activity of the patient [14,15]. Several studies have shown that this index reflects the parasympathetic response to noxious events during a painful stimulation [16–18]. However, to our knowledge no study has evaluated the effectiveness of ANI in evaluating pain in critically ill patients.

The primary aim of this prospective observational study is to evaluate the effectiveness of ANI in detecting pain in sedated, non-communicative, critically ill patients. The secondary aim was to evaluate the impact of norepinephrine use on ANI effectiveness, and to determine the correlation between ANI and BPS.

## Patients and Methods

### Settings and ethical aspects

This prospective observational study was conducted in two French medical ICUs (Lille University Hospital, and Victor Provo Hospital in Roubaix) during a 6-month period. The ethical committee of the *Société de Réanimation de Langue Française* approved the study (CE SRLF 11–239). The Institutional Review Board approved the study, and waived informed consent because of the non-interventional design. However, patients and/or their proxies were informed about the participation in this study.

### Inclusion and exclusion criteria

All deeply sedated adult patients, receiving invasive mechanical ventilation, and admitted to one of the two participating ICUs were eligible for this study. Exclusion criteria were light sedation, allowing communication with the patient; non-sinus cardiac rhythm; presence of pacemaker; atropine or isoprenaline treatment; and major cognitive impairment (massive stroke, resuscitated cardiac arrest).

### ANI computation process

The ECG signal is acquired at a 250 Hz sampling rate, according to published recommendations [19]. ECG is then analyzed using an automatic R wave detection algorithm in order to obtain the R-R intervals time series. Erroneous R wave detection and ectopic beats are filtered using a non linear artifact removal algorithm [20] and filtered R-R series are re-sampled at a 8 Hz frequency using a linear interpolation as recommended [19]. The re-sampled R-R series are normalized in a 64 seconds moving window for inter subject comparability. Normalization process includes two steps. First, the mean value (M) is computed as:

$$\text{M}=\frac{\text{1}}{\text{N}}\overset{\text{N}}{\underset{\text{i}=\text{1}}{\sum }}{\text{(RR}}_{\text{i}}\text{)},$$

where RR_i_ represents the R-R samples values and N the number of samples in the window. M is then subtracted from each sample of the window as: RR’_i_ = (RR_i_ − M).

Second, the norm values (S) are computed as:

$$\text{S}=\sqrt{\sum_{\text{i}=\text{1}}^{\text{N}}{\left({\text{RR'}}_{\text{i}}\right)}^{\text{2}}},$$

and each RR’_i_ is divided by S: RR”_i_ = RR’_i_ / S.

The normalized RR” series are then high pass filtered between 0.15 and 0.5 Hz, using a 4 coefficient Daubeuchies wavelet based filter.

**Fig 1:**
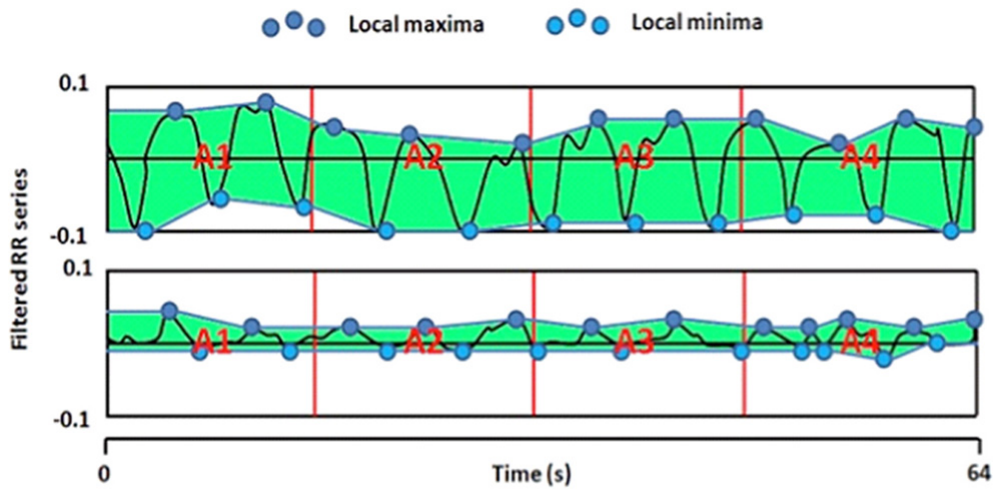
Mean centered, normalized and band pass-filtered RR series in 2 different levels of pain: without any painful stimulus (upper panel), under painful stimulus (lower panel).

Local maximum and minimum are detected on this filtered signal and the lower and upper envelopes are plotted in order to compute the areas between envelopes A1, A2, A3 and A4 in four 16 seconds sub windows. AUC_min_ is detected as the minimum value of A1, A2, A3 and A4 and ANI is defined as: ANI = 100 x (a xAUC_min_ + b) / 12.8, where *a* = 5.1, and *b* = 1.2 values have been determined empirically in a dataset of more than 200 R-R series analysis, in order to keep the coherence between the visual effect of parasympathetic influence on RR series and the quantitative measurement of ANI (Fig 1).

In clinical practice, ANI values close to 100 correspond to an important parasympathetic tone that can be associated to a high level of comfort. On the opposite, low ANI values are associated with a decreased parasympathetic tone, which is frequent in case of pain or anxiety.

### Physiological data acquisition

The ANI was recorded using the Physiodoloris^®^ monitor (MDoloris Medical Systems^®^, Loos, France). This monitoring device acquires the ECG signal through the analogical output of the patient’s multiparametric monitoring system, routinely used in the ICU and allowing a continuous display of ANI.

**Fig 2:**
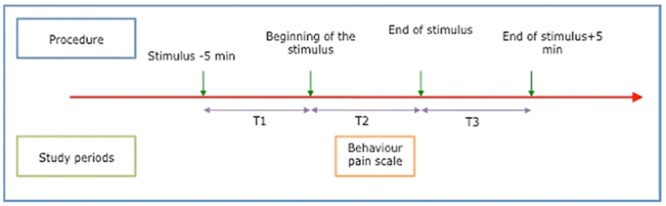
Time-points for ANI measurement.

In all patients, HR and ANI were continuously recorded during 5 minutes at rest (T1), during a painful stimulus (T2), and during 5 minutes after the end of the painful stimulus (T3) (Fig 2). The stimulus we chose was the turning of the patient for washstand. Previous studies showed that patient turning was one of the most painful nursing procedures [21]. Each patient was his own control, and could only be included once in the study. Pain was assessed at T2 by the BPS [22]. In order to not influence the measurement of the BPS, nurses and physicians were blinded to the ANI monitor.

### Study population and clinical data

Sedation was performed based on a nurse-driven protocol. Propofol and remifentanil were used for patients requiring short-term (<72h) sedation. Midazolam, and sufentanil were used for long-term sedation (≥72h). ATICE score was calculated every three hours to determine the depth of sedation and adjust infusion rate, based on the prescription of the attending physician, and the written protocol.

The following data were collected for all study patients: Simplified Acute Physiology Score II, and Logistic Organ Dysfunction score at ICU admission, depth of sedation according to the ATICE scale [23].

### Study Objectives

The primary objective was to demonstrate the effectiveness of ANI in detecting a painful stimulus in critically ill patients.

The secondary objectives were to determine the impact of norepinephrine on the effectiveness of ANI in detecting a painful stimulus, to evaluate the correlation between ANI and the BPS, and to determine factors associated with ANI during the painful stimulus.

### Statistical analysis

The expected mean ANI at T1 and T2 was 70% (SD 20%), and 50% (expected difference of 20%); respectively. Considering a power of 90%, and an alpha risk of 5%, the inclusion of 20 patients was required.

All statistical analyses were performed using SPSS 15.0 software. A p value <0.05 was considered significant. Qualitative variables are presented as numbers (percentage). The distribution of continuous variables was tested, using Shapiro Wilk test. These data are presented as median (interquartile range), because of their skewed distribution. Wilcoxon non-parametric test was performed to compare ANI values before, during and after the painful stimulus. Chi square, or Fischer exact test, and Mann Whitney non parametric test was used to compare qualitative, and quantitative data between patients with and without norepinephrine; respectively.

The correlation between ANI and BPS was analyzed using a Spearman correlation rank test. Correlation between ANI and other quantitative, and qualitative factors was evaluated using Spearman, and Pearson correlations; respectively. All factors with p<0.1 were included in a multiple linear regression model, using ANI as a dependent variable.

## Results

### Patient characteristics

**Fig 3:**
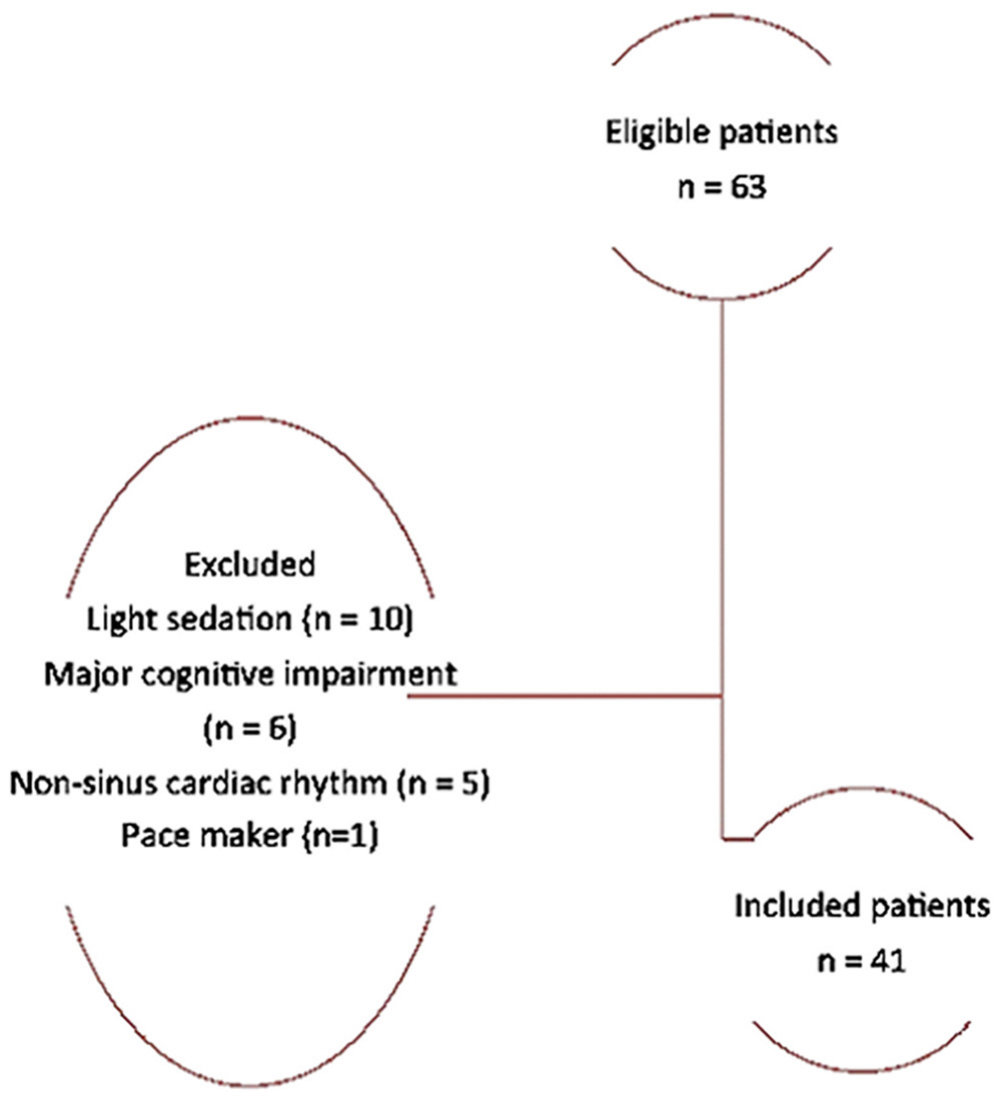
Study flowchart.

Forty-one patients were included, representing 65% of the 63 patients screened for eligibility. Twenty-two patients were excluded, including 10 for light sedation, 6 for major cognitive impairment, 5 for non-sinus cardiac rhythm, and 1 for pacemaker (Fig 3). Patient characteristics are presented in Tables 1 and 2. At ICU admission, Age, SAPS II, LOD score, respiratory, and hemodynamic failure were significantly higher in patients with norepinephrine compared with those without norepinephrine (Table 1). At ANI measurement, LOD score, and percentage of patients with sufentanil were significantly higher in patients with norepinephrine compared with those without norepinephrine. ATICE score was significantly lower in patients with norepinephrine compared with those without norepinephrine.

**Table 1:** Patient characteristics at ICU admission.

	Norepinephrine		
	No (n = 20)	Yes (n = 21)	P value
**Age**	58 (48–62)	70 (59–78)	0.004
**SAPS II**	53(41–60)	70 (56–79)	0.001
**LOD score**	8 (4–9)	10 (7–12)	0.018
**Cause for ICU admission**			
**Respiratory failure**	13 (65)	6 (28)	0.042*
**Hemodynamic failure**	4 (2)	17 (81)	<0.0001
**Neurologic failure**	4 (2)	2 (9)	0.410
**Others**	1 (5)	3 (14)	0.563
**Chronic comorbidities**			
**Diabetes**	2 (10)	6 (28)	0.238
**COPD**	8 (40)	4 (19)	0.141
**Cardiovascular disease**	2 (10)	2 (9)	1.000
**Cirrhosis**	1 (5)	1 (5)	1.000

SAPS, simplified acute physiology score; LOD, logistic organ dysfunction; COPD, Chronic obstructive pulmonary disease

Some patients had more than one cause for ICU admission.

Data are numbers (percentage), or median (interquartile range).

*OR (95% CI)4.7 (1.2–1.7)

**Table 2:** Patient characteristics at inclusion.

	Norepinephrine		
	No (n = 20)	Yes (n = 21)	P value
**LOD score**	6 (3–8)	10 (6–12)	0.003
**ATICE E**	2 (0–4)	1 (0.5–4)	0.759
**ATICE C**	0 (0–4)	0 (0–3)	0.929
**ATICE T**	10 (10–10)	8 (7–10)	0.003
**BPS**	5 (3–7)	6 (3–8)	0.277
**Neuromuscular blocking-agent use**	2 (10)	2 (9)	>0.999
**Midazolam**	12 (60)	15 (71)	0.659
**Propofol**	4 (20)	0 (0)	0.103
**Sufentanil**	6 (30)	15 (71)	0.019*
**Remifentanil**	11 (55)	6 (28)	0.162

LOD, logistic organ dysfunction; E, eyes; C, comprehension; T, tolerance; BPS, behavior pain scale

Data are numbers (percentage), or median (interquartile range).

*OR (95% CI)5.8(1.5–22)

### Effectiveness of ANI in detecting painful stimulus

**Fig 4:**
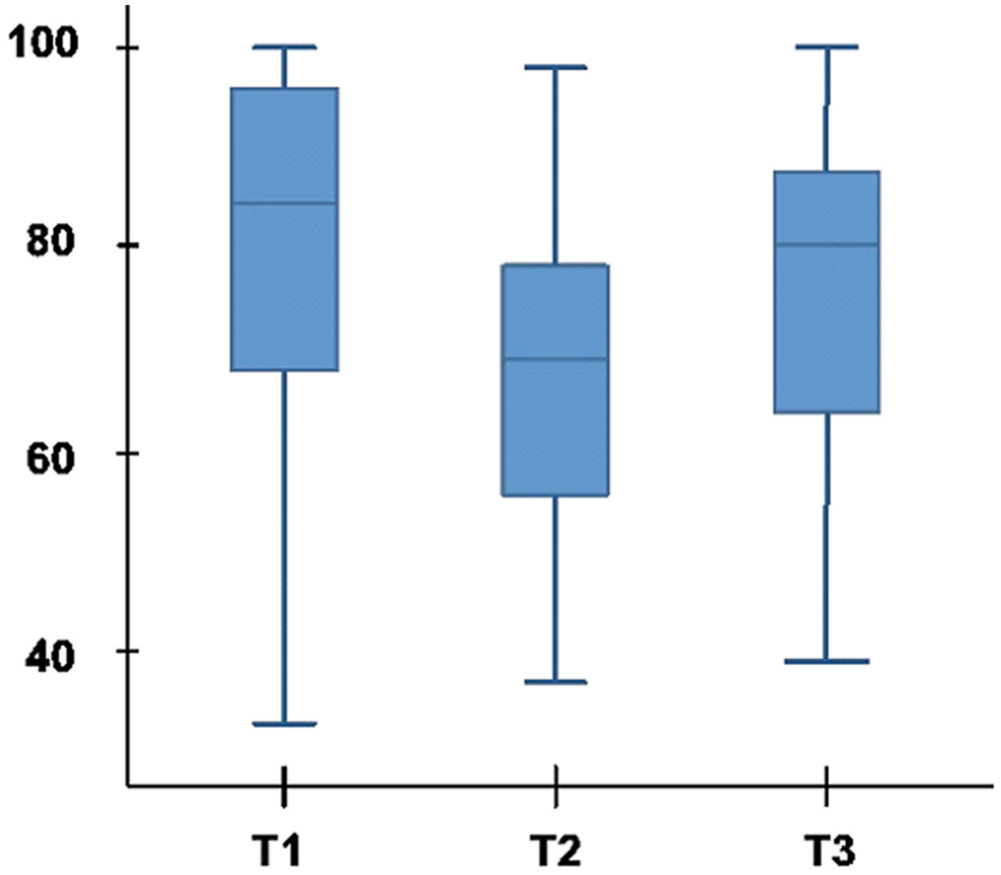
ANI values in study patients.

In all study patients, ANI was significantly lower at T2 (Med (IQR) 69(55–78)) than at T1 (85(67–96), p<0.0001), or T3 (81(63–89), p<0.0001) (Fig 4). No significant difference was found between ANI values obtained at T1, and T3.

In the subgroup of patients who did not receive norepinephrine (n = 20), ANI was significantly lower at T2 (57 (42–69)) than at T1 (74(66–80), p = 0.006), or T3 (68(53–88), p = 0.002).

In the subgroup of patients who received norepinephrine (n = 21), ANI was also significantly lower at T2 (75(66–80)) than at T1 (87(81–98), p = 0.001), or T3 (82(73–90), p = 0.023).

At T1 and T2, ANI values were significantly higher in the subgroup of patients who received norepinephrine, compared with those who did not receive norepinephrine. No significant difference was found in ANI values at T3 between these study subgroups (Table 3).

**Table 3:** ANI values in study subgroups.

	Norepinephrine		
	No (n = 20)	Yes (n = 21)	p
**ANI at T1**	74 (53–94)	87 (81–98)	0.043
**ANI at T2**	57 (42–69)	75 (66–80)	0.004
**ANI at T3**	68 (53–88)	82 (73–90)	0.072

T1, T2, T3: before, during and after the painful stimulus; respectively.

### Factors correlated with ANI during the painful stimulus

No significant correlation was found between ANI at T2 and BPS (p = 0.165, r^2^ = 0.221). In the subgroup of patients (n = 12) with BPS ≥ 7 (≥ 75^th^ quartile), no significant correlation was found between ANI at T2 and BPS (p = 0.944). Age (r^2^ = 0.48, p = 0.001), SAPS II (r^2^ = 0.38, p = 0.012), and dose of norepinephrine (r^2^ = 0.50, p = 0.001) were significantly correlated with ANI. No significant (p>0.05) correlation was found between ANI and all other variables (LOD score, cause for ICU admission, chronic comorbidities, sedation level, and dose of sedation). Multiple linear regressions showed a significant correlation between ANI and age (r^2^ = 0.43, p = 0.036).

## Discussion

Our results suggest that ANI is an effective tool to evaluate pain in deeply sedated critically ill patients. In addition, norepinephrine did not modify ANI effectiveness. However, no significant correlation was found between ANI and BPS.

### Study strengths and limitations

To our knowledge, our study is the first to evaluate a simple device for pain assessment in deeply sedated critically ill patients. The Physiodoloris^®^ device, used to assess ANI in this study, is totally noninvasive, easy to use, and compact. Using this device in the intensive care might be helpful to improve the quality of care, and reduce pain in critically ill patients.

However, some limitations of our study should be acknowledged. First, this study was observational, and included a small number of patients in two medical ICUs. Therefore, further large interventional studies are required to confirm our results in other populations, and to determine the value of this device in adjusting sedation level, improving patient comfort, and reducing unnecessary deep sedation. Second, the calculation of ANI is based on the respiratory sinus arrhythmia, which could be the result of pulmonary stretch receptors. However, several studies confirmed that this arrhythmia originated from the activity of bulbar respiratory centers [24]. Other studies evaluated whether mechanical ventilation, by reversing the intra-thoracic pressure regimen, reversed the respiratory sinus arrhythmia (i.e. induced a slowdown of cardiac frequency during insufflation). This was not confirmed, with important inter- and intra-individual variations, suggesting the importance of central nervous factors over mechanical influences [25]. However, in our study, each patient was used as its own control, which might have adjusted, at least in part, for these variations. Third, HRV might have occurred in response to the blood pressure variations, especially in patients with hemodynamic shock. However, these variations occur at low frequencies (basically 0,1 Hz), and are not taken into account in the ANI calculation. Fourth, ANI is a highly and quickly variable measure. For example, ANI falls immediately during the prick necessary for a capillary glycaemia, which is known to be a painful event. However, the continuous measurement of ANI allows clinicians to take its variability into account. Fifth, ANI was only evaluated during turning, and the impact of other painful procedures was not evaluated. However, turning critically ill mechanically ventilated patients is the most common nursing procedure in the ICU, and is one of the most painful procedures in these patients [2]. In fact, a recent study reported that pain defined as BPS score >3, or >5 was detected during turning in 94%, and in 64% of mechanically ventilated patients; respectively [26]. These results are in line with our findings, as median BPS value was 6 during tuning. Further, several previous studies clearly demonstrated the accuracy of ANI in detecting intraoperative, postoperative pain; as well as during labor, and experimentally induced pain [18,27–29]. Finally, ANI was evaluated only once per patient. However, it was recorded for 5 minutes during the painful stimulus, which might have allowed for brief variations in ANI values.

### Impact of norepinephrine on ANI measurement

The subgroup analysis demonstrated that ANI was effective in detecting pain in patients receiving norepinephrine. ANI values before, and during the painful procedure were significantly higher in patients receiving norepinephrine, compared with those not receiving norepinephrine. Because norepinephrine is a sympathomimetic drug, a decreased parasympathetic tone and a decrease in ANI values were expected in patients receiving norepinephrine. However, an elegant clinical study showed that parasympathetic-tone decrease, in response to norepinephrine use, was not constant [30]. Moreover, several studies reported that severe sepsis interferes with HRV, resulting in a reduction of the low frequency / high frequency ratio (i.e., a change in ANS balance) indicating an increased parasympathetic activity. A positive correlation was also found between plasmatic levels of pro-inflammatory cytokines in septic patients, and the increase of the “high frequency” component of HRV, which is related to the parasympathetic tone measured by the ANI. Another potential explanation for this result is the significantly deeper sedation, as shown by the lower ATICE score in patients who received norepinephrine compared with those who did not receive norepinephrine.

### Comparison of ANI with the BPS

We found no significant correlation between ANI and BPS. The recent clinical practice guidelines for the management of pain, agitation, and delirium in adult patients in the intensive care unit stated that both BPS, and CPOT demonstrated sufficient validity, and reliability to assess pain in critically ill patients, not able to communicate. One potential explanation for the absence of correlation between ANI and BPS is that ANI can detect painful events that do not result in modifications in the clinical signs included in this score. In other words, ANI is capable of detecting less intense pain. Absence of correlation can also be explained by the fact that ANI does not only measure pain, but also evaluates the neuropsychological dimension of pain, such as stress and discomfort. A recent study recorded ANI values in healthy volunteers before, during and after the display of a violent movie scene, considered as a negative emotional stimulus. The results showed a statistically significant decrease of ANI during the emotional stimulus [11]. Compared with the BPS, or to the CPOT, ANI has the advantage to be effective in paralyzed patients, and to provide instantaneous and continuous values. Moreover, this evaluation is totally objective, unlike all other available pain scales. Another potential explanation for the absence of significant correlation between ANI and BPS is the fact that patients were heavily sedated, suggesting that BPS values might not be accurate in this population. We performed a sensitivity analysis in only patients with high BPS score (≥75^th^ quartile), and did not find a significant correlation between ANI and BPS. This result could be explained by the small number of patients in this subgroup (n = 12).

## Conclusion

ANI is an effective tool to evaluate pain in deeply sedated critically ill patients. In addition, ANI is accurate for pain assessment in the subgroup of patients receiving norepinephrine. Measurement of ANI is a potentially interesting method to improve comfort in critically ill patients.

## Abbreviations

ANI: analgesia nociception index
ANS: automatic nervous system
BPS: behavioral pain scale
CPOT: critical care pain observatory tool
HR: heart rate
HRV: heart rate variability
